# Salt stress shapes rhizosphere bacterial communities and is associated with salt tolerance in tiger nut (*Cyperus esculentus* L.)

**DOI:** 10.3389/fpls.2026.1965745

**Published:** 2026-10-29

**Authors:** Gongfu Shi, Qi Dong, Jing Fang, Shaofeng Su, Rui Xie, Shuli Wei, Huimin Shi, Xinhui Huang, Zhanyuan Lu, Xiaoqing Zhao

**Affiliations:** 1 Inner Mongolia Academy of Agricultural and Animal Husbandry Sciences, Key Laboratory of Black Soil Protection and Utilization, Ministry of Agriculture and Rural Affairs, State Key Laboratory of Agricultural Ecology in the Agro-pastoral Ecotone (Cultivation), Inner Mongolia Key Laboratory of Degradation Farmland Ecological Restoration and Pollution Control, Hohhot, China; 2 School of Life Science, Inner Mongolia University, Hohhot, China; 3 College of Agronomy, Inner Mongolia Agricultural University, Hohhot, China

**Keywords:** *Cyperus esculentus* L., osmotic adjustment, plant–microbe interaction, rhizosphere bacteria, salt stress

## Abstract

**Background:**

Although soil salinization severely restricts crop production, plants can enhance their salt tolerance through interactions with rhizosphere microorganisms. Tiger nut (*Cyperus esculentus* L.) is a grain and oil plant characterized by its strong stress resistance. However, its salt tolerance mechanisms and their relation to its associated rhizosphere microorganisms remain unclear.

**Methods:**

This study involved a pot experiment with six NaCl salt concentrations (0, 0.36, 0.72, 1.08, 1.44, and 1.80 M). Through physiological and biochemical analyses, 16S rRNA sequencing, PICRUSt2 functional prediction, and partial least squares structural equation modeling (PLS-SEM), we explored the coupling relationships between the physiological responses of tiger nut and the succession of rhizosphere bacterial communities under a salt stress gradient.

**Results:**

The results showed that salt stress significantly increased the content of soluble sugar (SS), proline (Pro), and soluble protein (SP) in tiger nuts; enhanced the activity of superoxide dismutase (SOD) and catalase (CAT) (P < 0.05); and was associated with a shift in biomass allocation toward tuber development. Salinity is the primary driving force behind the directional reshaping of rhizosphere bacterial communities. The relative abundances of Proteobacteria and Firmicutes significantly increased, and *Planococcus* and *Paracoccus* were significantly enriched. Variance partitioning analysis showed that plant osmotic adjustment factors explained the largest independent fraction of the variation in rhizosphere bacterial community structure. The correlation analysis results indicate that predicted bacterial functional pathways associated with salt tolerance were significantly positively correlated (P < 0.05) with soil carbon- and nitrogen-cycling functions, as well as plant stress resistance traits and tuber dry weight. The PLS-SEM analysis results suggest that plant osmotic adjustment substances and predicted bacterial metabolic functions exhibited statistically mediated associations in the pathways linking salt stress to bacterial function, and potentially halotolerant rhizobacteria to plant antioxidant systems, respectively.

**Conclusions:**

In summary, this study suggests a potential synergistic association between plant osmotic adjustment and rhizobacterial enrichment in tiger nut under salt stress. In particular, the accumulation of plant osmotic adjustment substances was associated with the enrichment of rhizobacterial genera with reported halotolerant potential, and these enriched bacteria were closely linked to plant stress resistance traits, potentially through their salt-tolerance metabolic pathways. These findings provide correlative evidence for the association between plant osmotic adjustment and the enrichment of rhizobacterial genera with reported stress-resistant potential, which may be linked to salt tolerance in tiger nut. However, direct evidence from root exudate profiling or microbial inoculation experiments is still needed to confirm these associations.

## 1 Introduction

Soil salinization is one of the most severe environmental challenges, posing a serious threat to global agricultural production and ecological security (Xiao and Zhou, 2023). The area of salinized soil currently exceeds 1.3 billion hectares worldwide, accounting for 10.7% of the global land area, and over 5 million hectares of farmland are abandoned annually due to salt stress (Ivushkin et al., 2019; Khasanov et al., 2023). Global climate change has led to a continuous increase in the frequency and duration of droughts, which is expected to further exacerbate soil salinity in arid and semi-arid regions. Coupled with unreasonable land use practices (such as excessive fertilization and the use of high-salinity irrigation water), this has led to the estimate that nearly 50% of the world’s arable land will be affected by soil salinization by 2050. The accumulation of soluble salts in soil can lead to poor soil physicochemical properties, reduced soil enzyme activity, and decreased soil fertility (Fan et al., 2023). In addition, the absorption of high amounts of sodium ions (Na+) from the soil by plants can significantly hinder their absorption of nutrients such as potassium (K), leading to nutrient deficiency and ion toxicity (Guo et al., 2022). The accumulation of soluble salts in plants can also disrupt the osmotic balance of the plasma membrane, damage the integrity of the cell membrane, and inhibit their photosynthetic capacity, resulting in reduced growth, development, and, ultimately, yield. Therefore, against the global backdrop of increasingly severe soil salinization, a deep analysis of plant salt tolerance adaptation mechanisms is crucial for enhancing crop salt tolerance and improving agricultural productivity (Xu et al., 2025).

Although a plant’s adaptability to salt tolerance is often attributed to its genetic differentiation, rhizosphere microorganisms—referred to as the second genome of plants—play a crucial role in plant adaptation to ecological environments and can be considered as a key factor affecting plant stress resistance (Berendsen et al., 2012). Plant growth-promoting rhizobacteria (PGPR) are widely recognized as important microorganisms for improving plants’ adaptability to saline–alkali environments (Girma et al., 2022). They can promote salt and alkali tolerance in plants through various pathways, such as mediating ionic homeostasis, secreting plant hormones, promoting cell osmotic accumulation, improving antioxidant activity, and enhancing nutrient absorption (Wang et al., 2023). Through long-term interactions with saline–alkali environments, plants have evolved various survival mechanisms—including shaping soil microbial communities that meet their growth needs—that are precisely regulated by the key abiotic factor of salinity/alkalinity (Yue et al., 2020). Plants in low-salt environments typically exhibit milder selection pressure on their rhizosphere microorganisms, leading to the enrichment of a broad spectrum of growth-promoting bacterial communities in the rhizosphere, thus maintaining high community diversity (Liu et al., 2025a). As the salt concentration increases, plant osmotic stress intensifies, leading to root metabolic reprogramming. Characteristic metabolites such as organic acids, sugars, and proline are secreted into the soil for the targeted recruitment of salt-tolerant functional microorganisms. This drives significant changes in the microbial community structure, typically resulting in a decrease in community diversity (Leng et al., 2025). Furthermore, this recruitment process is often unique to the host species. Studies have shown that salt-tolerant rice varieties (such as Changbai 9) are more effective in recruiting specific bacterial groups such as *Hydrogenophaga*, *Pseudomonas*, and *Aeromonas*, as well as fungi such as *Chaetomium*, compared to sensitive varieties. These recruited groups become keystone taxa in the symbiotic network, enhancing the stability of the rhizosphere microbial network and reducing the Na^+^/K^+^ ratio in plant roots, thus promoting the rice plant’s resistance to salt stress (Lei et al., 2025). Therefore, as key environmental drivers of selection pressure, the intensity of salinity and the associated ion composition directly affect the composition and diversity of plant-shaped rhizosphere microbial communities. Therefore, analyzing the succession patterns of plant rhizosphere microbial communities under salinity gradients and the regulatory mechanisms of host–microorganism interactions are important scientific prerequisites for revealing the synergistic adaptations of plants and microorganisms to saline environments.

*Cyperus esculentus* L., commonly known as tiger nut, is an annual herbaceous plant belonging to the genus *Cyperus* in the family Cyperaceae. It is a multi-purpose crop used for grain, oil, and forage production. Characterized by its extensive root system, remarkable stress resistance, and high biomass production, this plant is of considerable strategic importance for the exploitation and sustainable use of saline soils in China (Diao et al., 2024). Studies have shown that saline tolerance in tiger nut exhibits inter-varietal differences, and its growth is significantly inhibited when the soil NaCl concentration exceeds 1.0 g/kg. In addition, the composition of the rhizosphere bacterial communities associated with tiger nut is significantly affected by salt stress. Re-inoculation with salt-tolerant strains isolated from the rhizosphere of tiger nut, such as Bacillus and Exiguobacterium, has been shown to effectively improve the germination rate, seedling height, and biomass of tiger nut plants under salt stress conditions, indicating that rhizosphere microorganisms play an important role in modulating the salt tolerance of this plant (Ying et al., 2026).

Although previous research has elucidated the physiological response characteristics of tiger nut under saline conditions and verified the growth-promoting potential of single functional bacterial strains, existing studies have mostly been limited to the determination of static physiological indicators and the screening and verification of single growth-promoting bacteria species. As such, there remains a lack of systematic analyses regarding the dynamic assembly patterns of rhizosphere microbial communities under continuous salinity gradients in tiger nut; the core driving factors mediating rhizosphere microbial differentiation; and the contributions of characteristic rhizosphere microorganisms and core bacterial communities to the salt tolerance traits of tiger nut under different salinity levels.

Therefore, based on the above-mentioned considerations, this study proposes the following scientific hypotheses: (1) changes in salinity gradients affect the accumulation of osmotic adjustment substances, such as proline and soluble sugars, in tiger nut leaves; (2) osmotic adjustment substances are key factors driving structural differences in the rhizosphere microbial communities of tiger nut; and (3) salt stress can cause metabolic reprogramming of microorganisms in the tiger nut rhizosphere, and these reprogrammed metabolic pathways are significantly correlated with the stress resistance traits of tiger nut.

To test these hypotheses, a gradient of salt treatments was established to characterize the dynamic succession patterns of rhizosphere microbial communities, explore the driving roles of plant metabolites in community assembly and, through functional prediction, reveal the potential mechanisms by which key bacterial taxa contribute to salt tolerance, in addition to quantifying their relative contributions to the formation of salt tolerance traits in tiger nut.

## 2 Materials and methods

### 2.1 Study site and experimental design

The field experiment was conducted at the experimental station of the Inner Mongolia Academy of Agricultural and Animal Husbandry Sciences (40°45′ N, 111°40′ E; 1040 m above sea level). The study area is characterized by a typical continental climate on the Mongolian Plateau, with a mean annual temperature of 6.7 °C, an average annual rainfall of about 390.7 mm, and a frost-free season ranging from 113 to 134 days. The experiment was conducted using the pot cultivation method, with a flower pot diameter of 27.5 cm, a bottom diameter of 22 cm, and a pot height of 31 cm. Each pot was filled with 13 kg of air-dried sandy soil. The tested sandy soil had an organic carbon content of 3.74 g kg^-^¹, available potassium of 26.45 mg kg^-^¹, available phosphorus of 18.65 mg kg^-^¹, and a pH of 8.73. The tested tiger nut variety was ‘Spain No. 1’, which is the main cultivated variety in central Inner Mongolia.

Salt stress treatments were designed according to the salinity distribution patterns observed in saline soils of Inner Mongolia. Previous studies have reported that soil salinity in the Hetao Plain of Inner Mongolia varies considerably, ranging from 0.6 to 17.5 g kg^-^¹, with the northwestern region exhibiting particularly severe salinization exceeding 10 g kg^-^¹ (Wang et al., 2016; Zhao et al., 2023). Six treatments were established: five NaCl concentration levels at 0.36 M (CC1), 0.72 M (CC2), 1.08 M (CC3), 1.44 M (CC4), and 1.80 M (CC5), with distilled water as the control (CK). Each treatment had three replicates, for a total of 18 pots. In this experiment, 2 L of NaCl solution at each concentration was applied to 13 kg of air-dried sandy soil. Based on the amount of NaCl added, the calculated final soil salt concentrations for the six treatments were 0, 3.24, 6.48, 9.72, 12.96, and16.2 g kg^-1^, respectively, giving a range of approximately 0-16.2 g kg^-1^. This range falls within the documented salinity range of Inner Mongolia and covers most of its salinization levels, and is therefore agronomically relevant to agricultural production in this area. Soil relative water content was maintained at around 60% of field capacity by the gravimetric method, and water was replenished every 2 days according to the weight loss. In preliminary trials, leaf margin yellowing and wilting were observed in tiger nut plants under the highest salt treatments after 18–20 days of salt exposure, which served as the sampling indicator. To prevent osmotic shock caused by the instantaneous application of salt solution, the required salt solution (2 L per pot) was split into 10 aliquots of 200 mL and applied sequentially, whereas the control plants received equal volumes of distilled water in parallel.

Twenty-five tiger nut seeds were uniformly sown per pot, and all pots were maintained in an outdoor rain-sheltered greenhouse at the experimental station. At 8–10 days after emergence, when approximately 80% of the seedlings had reached the five-leaf stage, the seedlings were thinned to 15 plants per pot, keeping those of uniform size and vigor.

After 17–19 days of emergence, 80% of the tiger nut seedlings entered the 7-leaf stage, and a salt solution was gradually applied for salt stress treatment until the plants in the high-salt treatment group showed symptoms of yellowing and wilting at the leaf edges, at which point samples were collected.

### 2.2 Sample collection and determination

#### 2.2.1 Plant sample collection and determination

For each treatment, three pots were set as three independent biological replicates. Three representative tiger nut plants were randomly selected from each pot as subsamples, and the average value of the three plants in the same pot was taken as one valid biological replicate data for this pot. Plant height was measured from the stem base to the leaf tip using a tape measure, and the average value was recorded. Soil Plant Analysis Development (SPAD) values were determined using a chlorophyll meter (TYS-A) at the middle portion of the leaf, with three readings taken per leaf. After these measurements, the roots and tubers were carefully excavated, gently rinsed with sterile distilled water, and blotted dry with filter paper. Fresh weights of roots and tubers were recorded separately. The samples were then placed in an oven at 105 °C for 30 min to deactivate enzymes, followed by drying at 80 °C until constant weight was achieved. The dry weights of roots and tubers were weighed and recorded separately.

Three representative plants were randomly selected from each pot, resulting in a total of nine subsamples per treatment. Functional leaves of the three plants from the same pot were fully mixed to form one composite sample, generating three independent biological samples per treatment (n=3). The leaves were rinsed with sterile distilled water, blotted dry with absorbent paper, and placed into 15-mL centrifuge tubes. Samples were immediately frozen in liquid nitrogen and stored at -80 °C in an ultra-low temperature freezer until analysis for antioxidant enzyme activities and osmotic adjustment substances. Soluble sugar (SS), proline (Pro), and soluble protein (SP) are widely used as physiological indicators associated with osmotic adjustment in plant stress physiology studies. Extensive research has demonstrated that soluble protein, together with soluble sugars and proline, serves as an important osmotic adjustment substance in plants, playing a critical role in maintaining cellular osmotic potential and protecting cell membrane integrity (Zong et al., 2023; Muhammad et al., 2025; Wen et al., 2026). Specifically, catalase (CAT) activity was determined by ultraviolet spectrophotometry; superoxide dismutase (SOD) activity by the NBT photochemical reduction method; peroxidase (POD) activity by the guaiacol colorimetric method; malondialdehyde (MDA) content by the thiobarbituric acid (TBA) colorimetric method; SS content by the anthrone colorimetric method; SP content by the Coomassie brilliant blue staining method; and Pro content by the ninhydrin colorimetric method (Akhoundian et al., 2025; Gu et al., 2025; Zhang et al., 2025).

#### 2.2.2 Soil sample collection and determination

Soil sampling was performed after leaf sample collection. Three representative plants were selected from each pot (nine subsamples in total per treatment). The complete root systems were carefully excavated, and the soil adhering to the roots was removed and sieved through a 1-mm mesh. Soil obtained from the three plants in the same pot was fully homogenized to form one independent biological soil sample. Each homogenized soil sample was divided and placed into two separate self-sealing bags for subsequent analysis of soil enzyme activities and microbial biomass, respectively. Rhizosphere soil tightly attached to the root surface of the three plants in each pot was collected using a sterile brush, sieved through a 1 mm mesh, and thoroughly mixed to form one independent biological rhizosphere sample per pot. The homogenized rhizosphere soil was aliquoted into 5 mL centrifuge tubes. All rhizosphere soil samples were immediately frozen in liquid nitrogen for subsequent rhizosphere microbial community diversity analysis. Soil catalase (S-CAT) activity was determined by ultraviolet spectrophotometry, urease activity by the indophenol blue colorimetric method, and alkaline phosphatase (ALP) activity by the disodium phenyl phosphate colorimetric method. Soil microbial biomass carbon, nitrogen, and phosphorus were determined according to the method of Jenkinson et al. (2004).

The extraction of total genomic DNA from soil microbial communities should refer to the instructions provided with the DNeasy PowerSoil^®^ Pro Kit (QIAGEN, Germany). The integrity of DNA was detected using 1% agarose gel electrophoresis, and the purity and concentration of DNA were measured using a NanoDrop 2000 ultra-micro spectrophotometer (Thermo Fisher Scientific, USA). The variable region of the bacterial 16S rRNA gene V3-V4 was selected for amplification, using primer sequences 338F (ACTCCTACGGGAGGCAGCAG) and 806R (GGACTACHVGGGTWTCTAAT). The amplification products were subjected to high-throughput sequencing on the Illumina MiSeq PE300 sequencing platform provided by Majorbio Biotechnology Co., Ltd. (Shanghai, China). Raw sequencing reads were subjected to quality control and filtering using fastp (version 0.20.0), followed by paired-end assembly with FLASH (version 1.2.7). The resulting clean reads had a length of approximately 415 bp. A total of 1,115,672 raw reads were obtained, and 965,427 reads were retained after quality filtering, with a retention rate of 86.53%. The average sequencing depth per sample was 53,635 reads. Then, using UPARSE software (http://drive5.com/uparse/, version 7.1), operational taxonomic units (OTUs) were clustered based on 97% sequence similarity (Edgar, 2013). A total of 5,969 OTUs were detected, and 1,326 OTUs were retained after rarefaction. To normalize sequencing depth, the read counts of all samples were rarefied to the minimum sequencing depth across samples. The sequencing coverage was 99.54%, and the rarefaction curve showsed that the number of OTUs reached saturation with increasing sequencing depth (**Supplementary Figure 1**), indicating that the current sequencing depth was sufficient to capture bacterial diversity. Taxonomic assignment of OTU representative sequences was performed using the Bayesian classifier (Classifier v2.2) against the SILVA database with a confidence threshold of 0.7. OTUs annotated as plant mitochondria or chloroplasts were removed (Wang et al., 2007). All downstream microbiome data analyses were conducted on the Majorbio Cloud Platform. Detailed sequencing data statistics are provided in **Supplementary Table 1**. The raw sequencing data have been deposited in the NCBI SRA database under BioProject accession number PRJNA1498546.

### 2.3 Statistical and bioinformatics analysis

Experimental data were organized using Excel 2021, and SPSS software (version 22) was employed for variance analysis (ANOVA) to assess the significance of inter-group differences (p<0.05 was considered significant). Prior to ANOVA, data normality was assessed using the Shapiro-Wilk test, and homogeneity of variance was assessed using Levene’s test. All indicators met the assumptions of normality and homogeneity of variance (P > 0.05). Given the small sample size per group (n = 3), Q-Q plots were also examined as a complementary visual assessment. For all multiple comparisons of plant physiological and biochemical indicators, one-way ANOVA followed by Tukey’s HSD *post-hoc* test was used to identify significant differences among treatments. For bacterial community composition, differences in the relative abundance of bacterial phyla were assessed using the Kruskal-Wallis H test. GraphPad Prism 8 was used to generate bar charts. The microbial α diversity index was calculated based on QIIME (v.1.9.1) software (Caporaso et al., 2010), and the Kruskal-Wallis H test was used to evaluate the differences in diversity indices between non-salt stress conditions. Principal coordinate analysis (PCoA) based on the Bray-Curtis distance matrix was calculated using the R package vegan (v2.5) to visualize the similarity of microbial community structure among samples. In addition, permutational multivariate analysis of variance (PERMANOVA) was applied to test the statistical significance of community structure differences among groups. Correlation network heatmaps and correlation heatmaps were visualized using the online platforms Microbiome Analyst (https://www.bioinformatics.com.cn) and OmicStudio (https://www.omicstudio.cn/tool), respectively. Species composition analysis, linear discriminant analysis effect size (LEfSe; LDA threshold > 4.0), variance partitioning analysis (VPA), Functional Annotation of Prokaryotic Taxa (FAPROTAX), and bacterial community functional prediction using PICRUSt2 were all performed on the Majorbio Cloud Platform. PICRUSt2 was used to predict KEGG Orthology (KO) abundances. The subsequent grouping of KOs into functional categories related to osmotic stress (KO_OS), antioxidative stress (KO_AS), ion transport (KO_IT), and nitrogen metabolism (KO_NIr) was performed manually by the authors based on KEGG functional annotations, gene/protein names, and published literature. Detailed information on each KO, including its annotation, KEGG pathway, grouping basis, and supporting references, is provided in **Supplementary Table 2**.

A partial least squares structural equation model (PLS-SEM) was constructed using SmartPLS 4.0. In this model, salt stress intensity was set as the exogenous latent variable, rhizosphere microbial community abundance as the mediating latent variable, and salt-stress tolerance traits of tiger nut as the endogenous latent variable. The significance of path coefficients was tested using 5,000 bootstrap resamples to verify the mediating role of rhizosphere microorganisms between salt stress and plant salt-tolerance traits. Complete measurement and structural model assessment results are provided in **Supplementary Tables 3**-**8**.

The Benjamini-Hochberg FDR procedure was applied to correlation analyses and Mantel tests; FDR-adjusted q-values are reported for these analyses. For PLS-SEM path coefficients and plant physiological/biochemical indicators, P values from bootstrap resampling and Tukey’s HSD are reported, respectively.

## 3 Results

### 3.1 Response characteristics of tiger nut to different salt stress levels

Salt stress significantly inhibited the vegetative growth of tiger nut, whereas moderate salinity was associated with a shift in biomass allocation toward tuber development. Compared with the control (CK), the salt treatments (CC) resulted in varying degrees of reduction in plant height (PH) and root dry weight (RDW), whereas tuber dry weight (TDW) significantly increased (**Figures 1A–C**). The reduction in plant height increased progressively with increasing salt concentration, with the lowest value (21.0 cm) recorded under CC5, representing a significant decrease of 50.44% compared with the control (CK) (P < 0.05; **Figure 1A**). For root dry weight, all treatments except CC1 and CC3 resulted in significantly lower values in comparison with CK (P < 0.05; **Figure 1B**). For root fresh weight (RFW), the CC3 treatment resulted in a significant increase compared with the other treatments (P < 0.05; **Supplementary Figure 2A**). Compared with CK, tuber dry weight (TDW) increased to varying degrees under salt stress, with the most pronounced increase observed under the CC3 treatment, where TDW reached 0.45 g—a value significantly higher than that of CK by 41.80% (P < 0.05; **Figure 1C**). These results indicate that salt stress not only inhibited the height of tiger nut plants, but also shifted biomass allocation toward tuber development.

**Figure 1:**
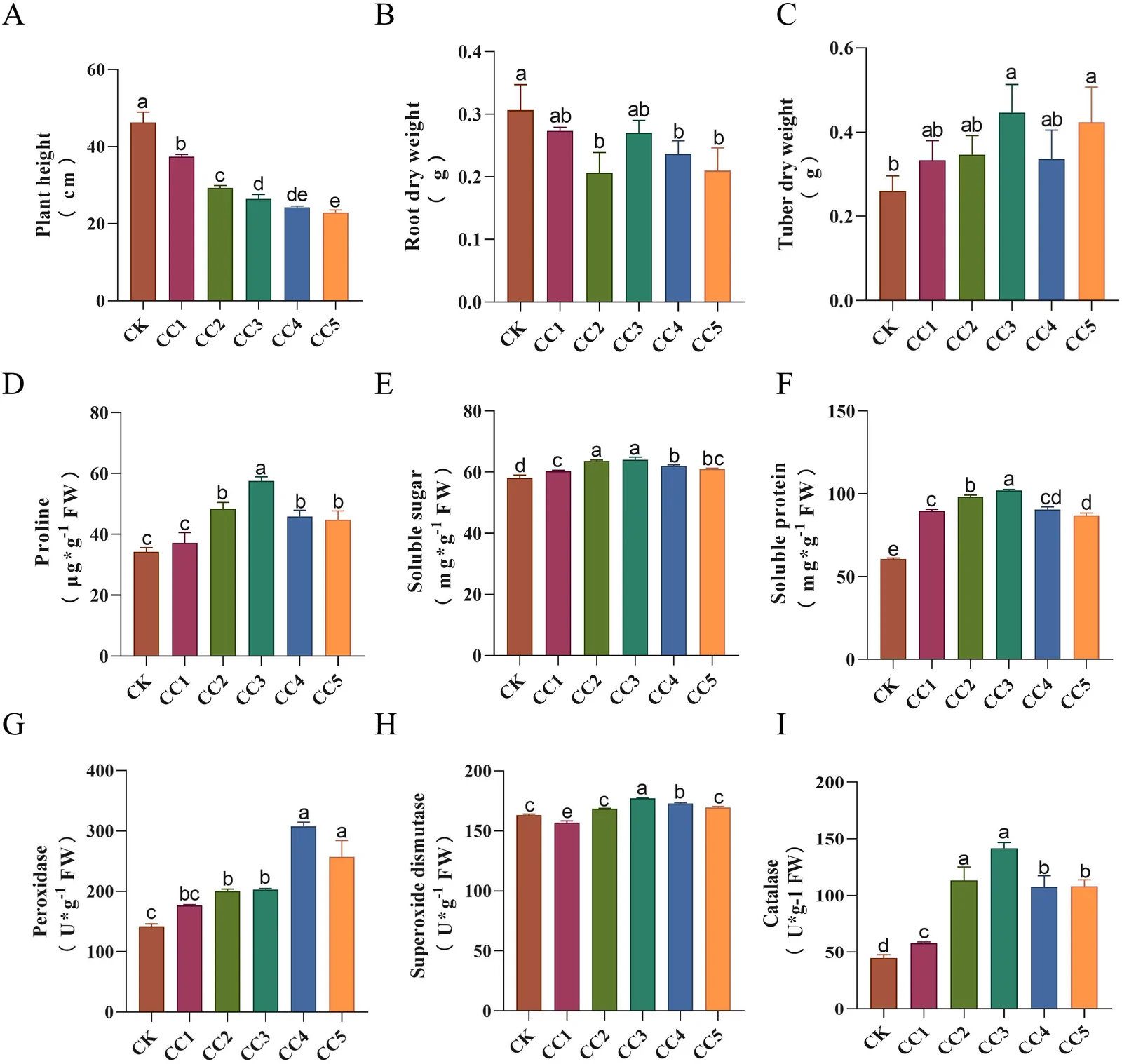
Effects of salt stress on growth and physiological characteristics of *Cyperus esculentus* L. **(A)** Plant height (PH); **(B)** Root dry weight (RDW); **(C)** Tuber dry weight (TDW); **(D)** Proline (Pro) content; **(E)** Soluble sugar (SS) content; **(F)** Soluble protein (SP) content; **(G)** Peroxidase (POD) activity; **(H)** Superoxide dismutase (SOD) activity; **(I)** Catalase (CAT) activity. CK, Control group (0 M NaCl); CC, Salt stress group (CC1, CC2, CC3, CC4, and CC5 represent 0.36 M, 0.72 M, 1.08 M, 1.44 M, and 1.80 M NaCl, respectively). Data are presented as mean ± standard deviation (SD) of three biological replicates (n = 3). Different lowercase letters above the bar graphs indicate significant differences between treatments (Tukey–Kramer test after one-way ANOVA, P < 0.05).

In terms of osmotic adjustment, salt stress significantly induced the accumulation of osmotic adjustment substances in tiger nut (P < 0.05; **Figures 1D–F**). Among them, the contents of proline, soluble sugar, and soluble protein were highest under the CC3 treatment when compared to other treatments, reaching 57.55 μg·g^-^¹ FW, 64.04 mg·g^-^¹ FW, and 101.94 mg·g^-^¹ FW, respectively (i.e., 40.46%, 9.20%, and 40.63% increases compared to CK, respectively). In terms of antioxidant defense, salt treatment significantly affected the antioxidant enzyme activity in tiger nut. Compared with the control (CK), the activity of peroxidase (POD) significantly increased under the salt stress treatments. The activities of superoxide dismutase (SOD) and catalase (CAT) showed a trend of first increasing and then decreasing with the salt concentration gradient, reaching maxima of 203.00 U·g^-^¹ FW and 141.83 U·g^-^¹ FW, respectively, at CC3 (P < 0.05; **Figures 1G–I**). The content of malondialdehyde (MDA) also exhibited differential responses under different salt stresses (**Supplementary Figure 2C**). There was no significant difference in the MDA level under the CC1 and CC2 treatments when compared to the control (CK), while its level was significantly increased under high salt stress (CC4, CC5) (P < 0.05). In addition, compared to the control (CK), the relative chlorophyll content (SPAD) also significantly decreased under high salt stress (CC5) (P < 0.05; **Supplementary Figure 2D**). These results indicate that tiger nut can resist a certain degree of salt stress damage by increasing the accumulation of osmotic adjustment substances and enhancing antioxidant enzyme activities. In this process, biomass allocation was shifted toward tuber development, which may represent an adaptive response to salt stress. Among the conditions tested in this experiment, CC3 (1.08 M NaCl) was found to be the most suitable concentration for inducing the physiological response to salt stress in tiger nut.

### 3.2 Responses of soil enzyme activities and microbial biomass to salt stress gradients in tiger nut

Salt stress significantly influenced soil enzyme activities and microbial biomass in the rhizosphere of tiger nut, with marked variations in the response patterns among individual parameters (**Figures 2A–F**; **Supplementary Figure 3**). In terms of soil enzyme activity, there were no significant differences in sucrase (SC) activity among the treatments (P > 0.05). Catalase (S_CAT) activity continued to increase with increasing salt concentration, showing increases in the range of 9.56%-16.85% when compared to the control group (CK) (**Figure 2A**). Both urease (URE) and alkaline phosphatase (ALP) activities significantly decreased under salt stress compared to the CK, with their greatest decreases reaching 17.27% and 31.95%, respectively (**Figures 2B, C**). In terms of soil microbial biomass-related variables, microbial biomass carbon (MBC), microbial biomass nitrogen (MBN), and microbial biomass phosphorus (MBP) all exhibited a significant downward trend under salt stress (P < 0.05). These results indicate that salt stress has differential effects on soil enzyme activities and microbial biomass regulation. S_CAT activity significantly increased, while URE and ALP activities—as well as microbial biomass variables—were significantly inhibited. This may reflect the soil microbial community’s differentiated adaptation strategies to salt stress, in terms of both structure and function.

**Figure 2:**
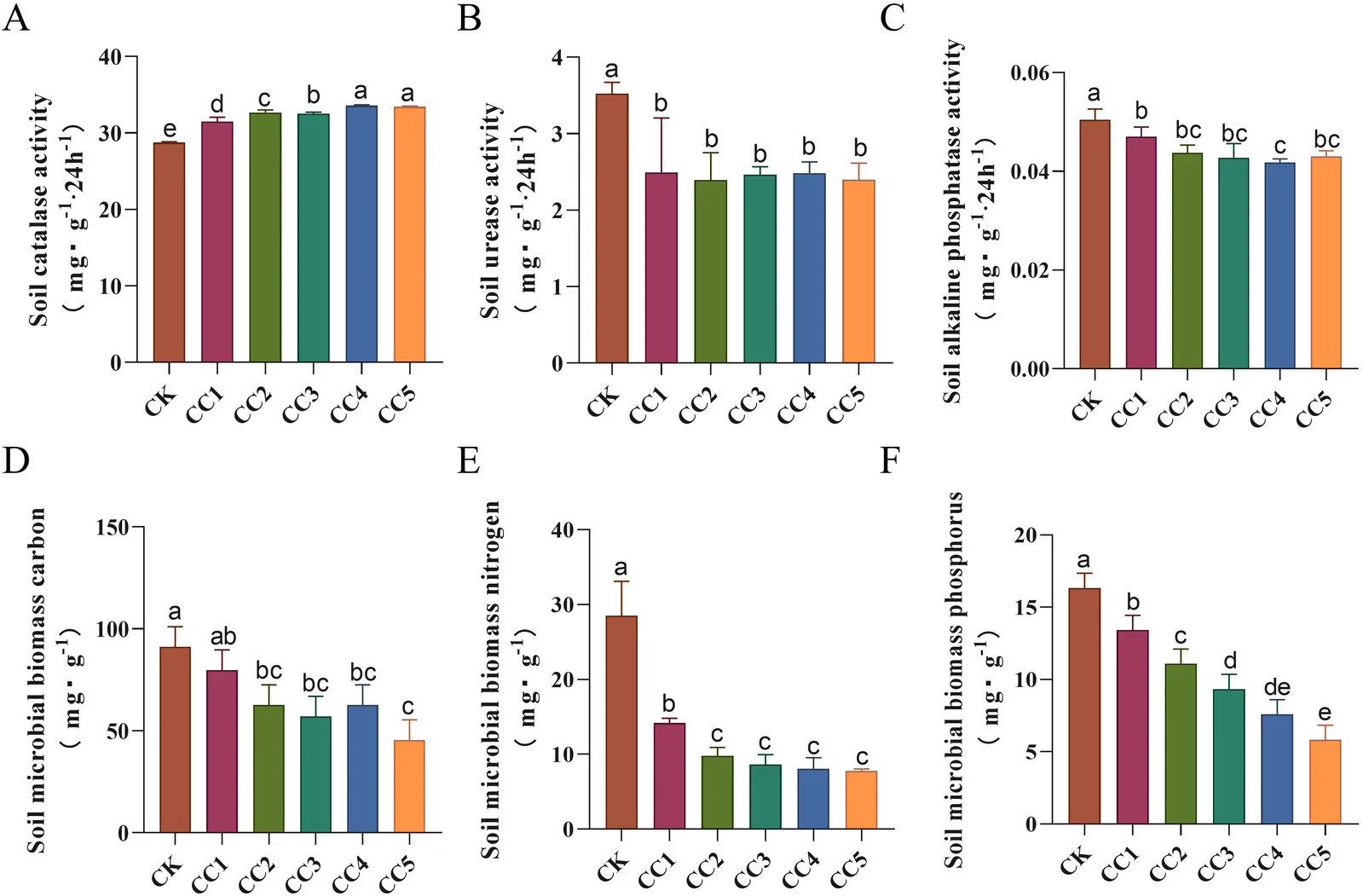
Effects of salt stress on soil enzyme activities and microbial biomass-related variables of tiger nut rhizosphere: **(A)** Catalase (S_CAT) activity; **(B)** Urease (URE) activity; **(C)** Alkaline phosphatase (ALP) activity; **(D)** Soil microbial biomass carbon (MBC); **(E)** Soil microbial biomass nitrogen (MBN); **(F)** Soil microbial biomass phosphorus (MBP). CK, control group (0 M NaCl); CC1-CC5, salt treatment groups with increasing NaCl concentrations. Data are presented as mean ± standard deviation (SD) of three biological replicates (n = 3). Different lowercase letters above the bars indicate significant differences among treatments (one-way ANOVA followed by Tukey–Kramer test, P < 0.05).

### 3.3 Characteristics of rhizosphere bacterial communities in tiger nut under different salt stress levels

Salt stress significantly altered the diversity and community composition of rhizosphere bacterial communities associated with tiger nut (**Figures 3A–G**). In terms of α-diversity, both the Shannon and Sobs indices exhibited a trend of decreasing first and then increasing with increasing salt concentration (**Figures 3A, B**). Compared to CK, these indices slightly increased under low-salt treatment (CC1), significantly decreased under CC2 treatment (P < 0.05), and reached the lowest level under CC3 treatment. Meanwhile, under high-salt treatments (CC4, CC5), both indices gradually rebounded. These results indicate that moderate salt stress can enhance the species richness and evenness of the rhizosphere microbial community; however, when the salt concentration reaches a certain threshold, sensitive groups are inhibited, leading to a decrease in community diversity. As salt stress further intensifies, salt-tolerant microorganisms dominate the bacterial population, and the richness and diversity of the community increase. In terms of β-diversity, Principal Coordinate Analysis (PCoA) based on the OTU level revealed a clear separation of the treatment groups along the first principal coordinate axis. Furthermore, through PERMANOVA analysis, significant differences in microbial community structure were identified among different salt treatments (R = 0.8494, P = 0.001; **Figure 3C**), indicating that salt concentration is the dominant factor driving the variation in the rhizosphere microbial community structure.

**Figure 3:**
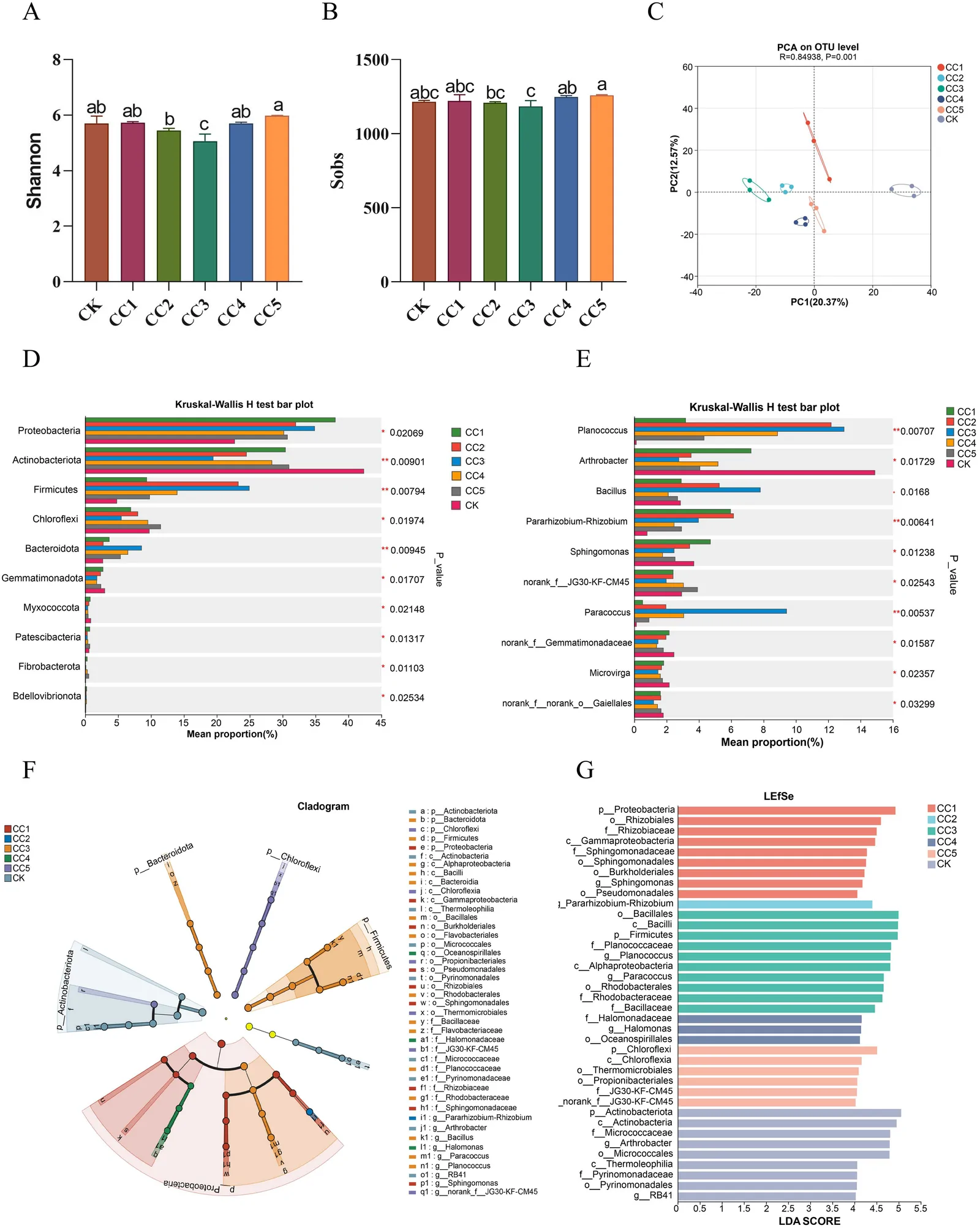
Effects of salt stress on soil microbial community structure of the *Cyperus esculentus* L. rhizosphere: **(A)** Shannon diversity index; **(B)** Sobs richness index; **(C)**. Principal coordinate analysis (PCoA) based on OTU level (R = 0.8494, P = 0.001); **(D)** Relative abundance of top 10 phyla (Pararhizobium–Rhizobium represents Allorhizobium–Neorhizobium–Pararhizobium–Rhizobium); **(E)** Relative abundance of top 10 genera; **(F)** Cladogram of LEfSe analysis, showing differentially abundant taxa from phylum to genus level; **(G)** Linear discriminant analysis (LDA) scores of differentially abundant taxa (LDA threshold = 4.0). CK, control group (0 mM NaCl); CC-CC5, salt treatment groups with increasing NaCl concentrations. Data are presented as mean ± standard deviation (SD) of three biological replicates (n = 3). Different lowercase letters above the bars indicate significant differences among treatments (Kruskal–Wallis H test followed by Dunn’s *post hoc* test, P < 0.05).

In terms of community composition at the phylum level, the top ten dominant phyla showed significant differences in distribution across various treatments (**Figure 3D**; **Supplementary Figure 4A**). Among them, Proteobacteria, Actinobacteria, and Firmicutes were the dominant phyla under all treatments. However, as the salt concentration increased, the relative abundance of Actinobacteria showed a downward trend, while that of Firmicutes first increased and then decreased, reaching a maximum under the CC3 treatment (4.88 times that of CK). At the genus level, *Planococcus*, *Paracoccus*, and *Allorhizobium–Neorhizobium–Pararhizobium–Rhizobium* all significantly increased under salt stress (**Figure 3E**; **Supplementary Figure 4B**). These genera showed the greatest increases under the CC3 treatment, being 104.70, 4.99, and 72.35 times higher than the control (CK), respectively. Furthermore, the relative abundance of *Bacillus* also exhibited a trend of initially increasing followed by a decrease with increasing salt concentration, also peaking at CC3 (2.73 times higher than the CK). These results indicate that genera such as *Planococcus*, *Allorhizobium–Neorhizobium–Pararhizobium–Rhizobium*, *Paracoccu*s, and *Bacillus* have a strong competitive advantage under salt stress.

To further identify indicator species with differences among the treatment groups, we conducted a LEfSe analysis. The cladogram (**Figure 3F**) displays the enriched taxa from the phylum to genus level across the treatment groups. Through LDA (with a threshold of 4.0), the contributions of the differential species were further quantified (**Figure 3G**). The results showed that the CK group was mainly enriched with *Arthrobacter* belonging to the phylum Actinobacteria; CC1 and CC2 were mainly enriched with Proteobacteria, among which *Sphingomonas* from the Sphingomonadaceae family and *Allorhizobium–Neorhizobium–Pararhizobium–Rhizobium* from the Rhizobiaceae family were the indicator species under the respective salt concentrations; the main bacterial groups in CC3 included Flavobacteriaceae from the phylum Bacteroidetes, *Bacillus* and *Planococcus* from the phylum Firmicutes, and *Paracoccus* from the phylum Proteobacteria; CC4 was dominated by *Halomonas* from the phylum Proteobacteria; and CC5 was mainly characterized by Actinobacteria Propionibacteriales and norank_f_JG30-KF-CM45. In summary, salt stress significantly reshapes the microbial community structure in the rhizosphere soil of tiger nut, with the diversity and composition of species undergoing systematic succession along the salt concentration gradient. The relative enrichment of genera *Planococcus*, *Paracoccus*, and *Halomonas* may reflect an adaptive response of the rhizosphere microbial community under salt stress conditions.

### 3.4 Key factors driving rhizosphere bacterial community assembly in tiger nut

To explore the key factors driving structural changes in the rhizosphere microbial community of tiger nut under salt stress, we performed Redundancy Analysis (RDA), Variance Partitioning Analysis (VPA), and correlation analyses linking soil properties, plant traits, and dominant bacterial genera (**Figures 4A–C**). The RDA results (**Figure 4A**) revealed that the selected soil and plant factors collectively explained 80.38% of the variation in rhizosphere microbial community structure (RDA1: 66.26%, RDA2: 14.12%). Moreover, URE, S_CAT, ALP, SP, SS, and Pro were located at relatively large distances from each other on the RDA ordination plot, indicating that salt stress-induced changes in soil microbial traits and plant physiological metabolism jointly drive reassembly of the microbial community.

**Figure 4:**
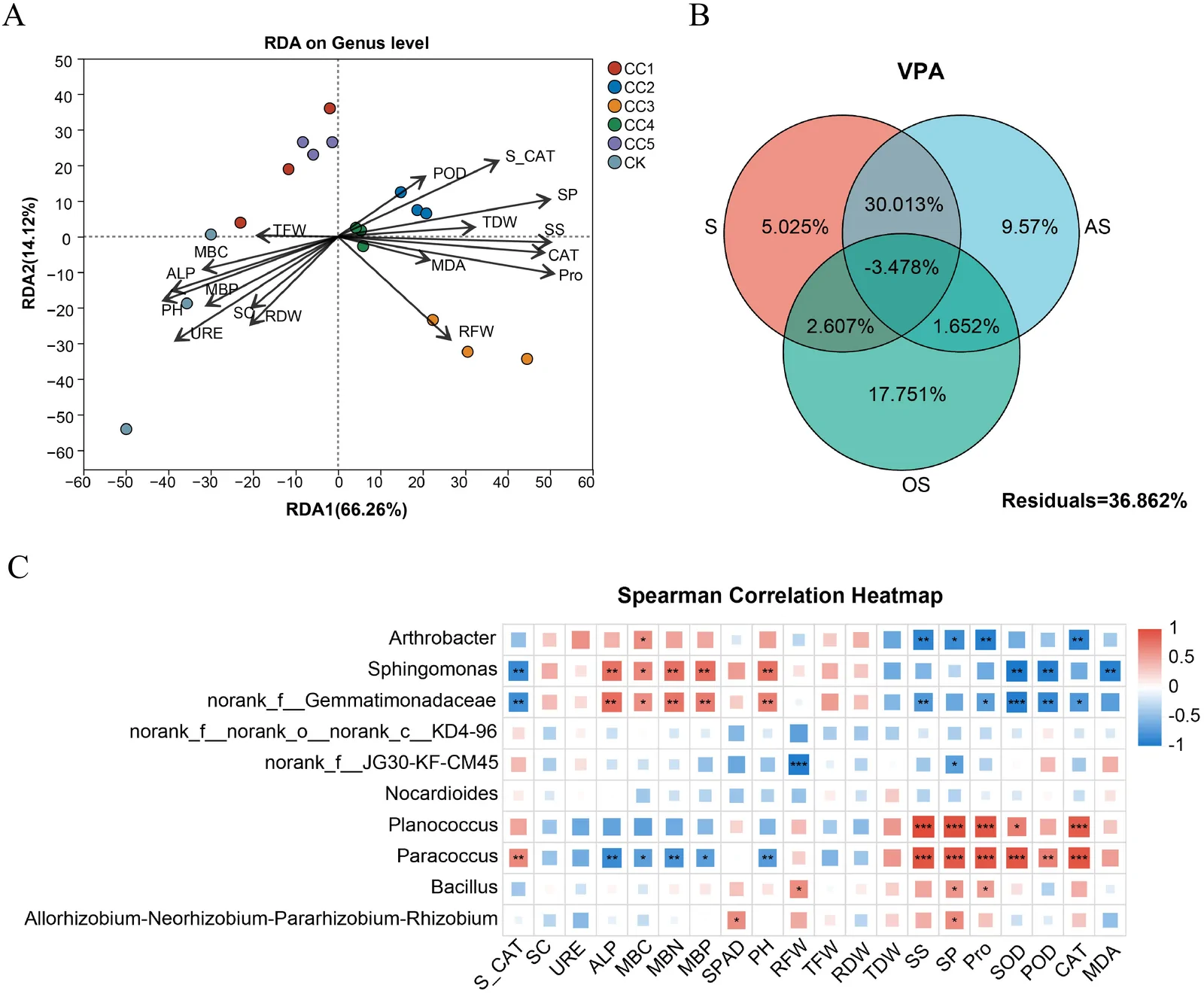
Key driving factors for the assembly of rhizosphere microbial communities under salt stress: **(A)** Redundancy analysis (RDA) of rhizosphere microbial community structure and environmental variables; **(B)** Variation partitioning analysis (VPA) quantifying the contributions of the plant antioxidant system (AS, including SOD, POD, and CAT), soil properties (S, including S_CAT, URE, ALP, MBC, MBN, and MBP), and plant osmotic regulation system (OS) to microbial community variation; **(C)** Spearman correlation heatmap between top 10 genera and environmental factors. S_CAT, soil catalase; URE, urease; ALP, alkaline phosphatase; MBC, microbial biomass carbon; MBN, microbial biomass nitrogen; MBP, microbial biomass phosphorus; SPAD, relative chlorophyll content; PH, plant height; RFW, root fresh weight; TFW, tuber fresh weight; RDW, root dry weight; TDW, tuber dry weight; SS, soluble sugar; SP, soluble protein; Pro, proline; SOD, superoxide dismutase; POD, peroxidase; CAT, plant catalase; MDA, malondialdehyde. All analyses were based on three biological replicates per treatment (n = 3). *q<0.05, **q<0.01, ***q<0.001.

Through VPA, the relative contributions of plant physiological metabolism and soil traits to community structure variation were further evaluated. In particular, plant physiological metabolism was characterized by SP, SS, Pro, CAT, POD, SOD, and MDA, while soil traits were characterized by S_CAT, URE, ALP, MBC, MBN, and MBP. The results indicated that the total constrained variation explained by all measured variables was 63.14% of microbial community variation (**Figure 4B**; **Supplementary Figure 5**), leaving 36.86% of the variation unexplained. The independent contribution of plant physiology and biochemistry (P) was 28.97%, while that of soil traits (S) was 5.03% (**Supplementary Figure 5**). To further dissect the contributions of plant traits to microbial community shifts, we categorized them into the antioxidant system (AS)—comprising CAT, POD, SOD, and MDA—and the osmotic adjustment system (OS)—comprising SP, SS, and Pro—and analyzed their contributions alongside those of soil traits (S). The results showed that the independent contribution of OS traits was the largest, reaching 17.75%, followed by AS, while the independent contribution of soil traits was the smallest, at 5.03%. Among the measured variables, the osmotic adjustment system exhibited the strongest independent association with microbial community variation.

The correlation analysis revealed that, after FDR adjustment, both *Planococcus* and *Paracoccus* remained extremely significantly positively correlated with plant SS (r = 0.9071, r = 0.8700), SP (r = 0.8766, r = 0.8384), Pro (r = 0.8533, r = 0.8543), SOD (r = 0.6591, r = 0.8526), and CAT (r = 0.8225, r = 0.8638) (q < 0.01). For soil properties, *Paracoccus* remained significantly positively correlated with S_CAT (r = 0.6201, q < 0.05) and significantly negatively correlated with ALP (r = −0.7139), MBC (r = −0.5868), MBN (r = −0.6588), and MBP (r = −0.5874) (q < 0.05), whereas the correlations between *Planococcus* and these soil properties did not remain significant after FDR adjustment (q > 0.05). This pattern suggests that plant osmotic adjustment may exert a stronger influence than soil properties on the enrichment of *Planococcus* and *Paracoccus*. In contrast, *Sphingomonas* and *norank_f_Gemmatimonadaceae* showed significant or extremely significant positive correlations with ALP (r=0.7253, r=0.7470), MBC (r=0.6240, r=0.5846), MBN (r=0.7269, r=0.7083), and MBP (r=0.7435, r=0.6753), and an extremely significant negative correlation with S_CAT (r=-0.7257, r=-0.6988). Additionally, they showed significant negative correlations with plant Pro (r=-0.5124, r=-0.5847), SOD (r=-0.8288, r=-0.7946) (q > 0.05) (**Figure 4C**; **Supplementary Table 9**), and POD (r=-0.7915, r=-0.7337), indicating that these two bacterial genera are regulated by oxidative factors and microbial biomass, with an oxidative environment having a greater impact.

In summary, the restructuring of the rhizosphere microbial community of tiger nut under salt stress was closely associated with the synergistic effects of plant physiological responses (antioxidant and osmotic regulation) and changes in the soil environment. Among these factors, the osmotic regulation substances of plants were identified as the primary factor associated with rhizosphere microbiota restructuring under salt stress. This suggests a potential linkage between plant osmotic adjustment substances and the enrichment of specific functional microorganisms, which may contribute to plant adaptability under salt stress.

### 3.5 Correlation analysis of rhizobacterial functions with plant stress resistance and biomass under salt stress

To analyze the relationships between salt stress-induced shifts in the rhizosphere bacterial community structure and plant salt tolerance, we first used FAPROTAX and PICRUSt2 to predict the functional composition of the rhizosphere bacterial community. We then explored the correlations among potentially halotolerant rhizobacteria, their salt-tolerance-related KO pathways, and the stress resistance and biomass traits of tiger nut via correlation analysis, Mantel tests, and partial least squares structural equation modeling (PLS-SEM) (**Figures 5A–F**).

**Figure 5:**
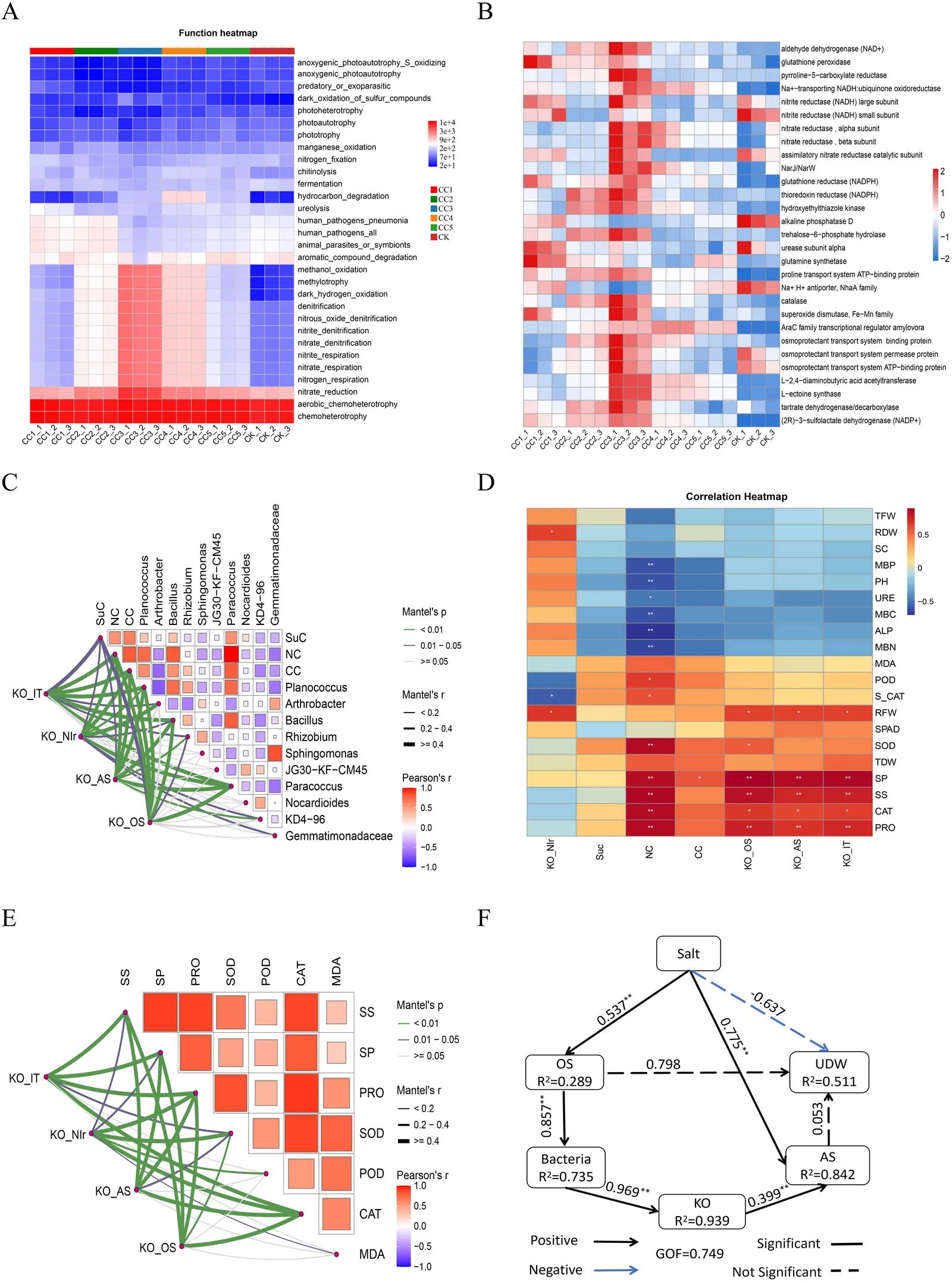
Functional shifts and host–microbe interactions of the rhizosphere microbiome under salt stress: **(A)** FAPROTAX-based functional prediction heatmap showing changes in carbon, nitrogen, and sulfur cycling-related functions. **(B)** PICRUSt2-predicted KO pathway heatmap for ion transport (KO_IT), osmotic substance synthesis (KO_OS), antioxidant enzymes (KO_AS), and nitrogen metabolism (KO_NIr). **(C)** Correlation heatmap among predicted KO pathways; FAPROTAX-predicted carbon (CC), nitrogen (NC), and sulfur (SuC) cycling functions; and dominant genera. **(D)** Mantel test-based association analysis between predicted KO pathways and functional traits. **(E)** Mantel test-based analysis of predicted KO pathways and plant physiological traits. *q<0.05; **q<0.01. **(F)** Partial least squares structural equation modeling (PLS-SEM) illustrating salt stress-driven interactions between *Cyperus esculentus* L. and rhizosphere bacteria. CK, control; CC1-CC5, salt treatments. All analyses were based on three biological replicates per treatment (n = 3). *P<0.05; **P<0.01.

The FAPROTAX functional prediction heatmap (**Figure 5A**) shows that salt stress significantly altered the predicted relative abundances of functions related to carbon, nitrogen, and sulfur cycling in the rhizosphere bacterial community. Chemoorganoheterotrophy and aerobic_chemoheterotrophy—both related to the carbon cycle—were dominant functions across all treatments. The predicted relative abundances of nitrogen-cycling functions, including nitrite_denitrification, denitrification, nitrate_denitrification, nitrate_respiration, and nitrogen_respiration, were significantly enriched (P < 0.05) under low- and medium-salt treatments (CC1-CC3), but gradually decreased under high-salt treatments (CC4, CC5). The predicted relative abundance of sulfur-cycling functions, specifically dark_oxidation_of_sulfur_compounds, was relatively low and showed an increasing then decreasing trend with increasing salt concentration. These results indicate that moderate salt stress promotes the overall activity of nitrogen-cycling and sulfur-cycling functions in rhizosphere bacteria, selectively enhancing specific metabolic pathways such as nitrification and nitrate reduction.

PICRUSt2 prediction was further performed to analyze the functional responses of bacteria under different levels of salt stress. The KO functional pathway heatmap indicates that functionally advantageous metabolic pathways are related to bacterial nitrogen metabolism (KO_NIr), salt ion transport (KO_IT), antioxidant enzyme system (KO_AS), and accumulation of osmolytes (KO_OS) (**Figure 5B**). The results demonstrate that predicted abundances of functions associated with KO_NIr (e.g., nitrite reductase), KO_AS (e.g., glutathione peroxidase), KO_IT (e.g., Na^+^-transporting NADH: ubiquinone oxidoreductase), and KO_OS (e.g., pyrroline-5-carboxylate reductase) were continuously higher under treatments CC1-CC3, reaching the highest level under CC3 and then decreasing. These results indicate that the predicted impacts of salt stress on the functioning of rhizosphere bacteria vary with the salt concentration; in particular, moderate salt stress was associated with higher predicted abundances of multiple metabolic pathways, while high salt stress selectively inhibited certain functions.

The Mantel test and correlation analysis results further revealed the association patterns between the predicted KO functional pathways and soil properties, plant traits, and dominant bacterial genera (**Figures 5C, D**). Regarding the associations between bacterial genera and their functions, the Mantel test indicated significant positive correlations after FDR adjustment (q < 0.05) between the predicted abundances of KO_NIr, KO_IT, KO_AS, and KO_OS and the sulfur cycle (SuC), nitrogen cycle (NC), and carbon cycle (CC). Notably, after FDR adjustment, *Planococcus* and *Paracoccus* showed extremely significant positive correlations with KO_NIr, KO_IT, KO_AS, and KO_OS, with Mantel correlation coefficients of r = 0.33 and 0.46, r = 0.74 and 0.76, r = 0.74 and 0.61, and r = 0.72 and 0.78, respectively (**Supplementary Table 10**). Thus, these two genera possess strong salt tolerance potential and may be associated with soil nutrient cycling through KO-mediated metabolic pathways. In the correlation analysis between the KO pathways and soil and plant traits, KO_IT, KO_AS, and KO_OS showed highly significant positive correlations with RFW, SP, SS, CAT, and Pro after FDR adjustment (all r values greater than 0.64, q < 0.01) (**Supplementary Table 11**), indicating that the higher predicted abundances of bacterial functional pathways were associated not only with plant stress resistance traits but also with plant root growth. The Mantel analysis of the KO pathways and plant stress resistance traits and biomass (**Figure 5E**) revealed significant positive correlations between KO_NIr, KO_IT, KO_AS, and KO_OS and plant SS, Pro, SP, SOD, and CAT after FDR adjustment (q < 0.05). These results indicate that the ion transport, osmotic regulation, antioxidant defense, and nitrogen-cycling functions of rhizosphere bacteria are closely related to plant stress resistance and biomass traits. They also suggest that bacteria may be associated with plant stress resistance traits and salt tolerance through these predicted metabolic pathways.

The PLS-SEM results further quantified the direct and indirect effects among salt stress, plant stress-resistance traits, and bacterial KO pathways potentially associated with salt tolerance. (**Figure 5F**). The results show that the direct effects of salt stress on the osmotic adjustment and antioxidant systems of plants were 0.537 and 0.775, respectively (P < 0.01); the direct effect of plant osmotic adjustment substances on dominant potentially halotolerant rhizobacteria in the rhizosphere was 0.857; and the direct effect of dominant potentially halotolerant rhizobacteria on salt-tolerance metabolic pathways was 0.969 (**Supplementary Table 12**). These results indicate that salt stress has a significant and positive direct effect on plant stress resistance traits, which can indirectly affect bacterial communities and functions through the influence of plant osmotic adjustment substances (P < 0.01). The mediation effect test revealed that the chain indirect effect of the plant osmotic adjustment system–dominant bacteria–bacterial salt-tolerant function pathway was 0.831, with a 95% confidence interval of 0.746-0.909, indicating a statistically significant mediation pathway linking plant osmotic adjustment to the salt-tolerance functional potential of rhizosphere bacteria through the enrichment of dominant potentially halotolerant taxa. Further path analysis revealed that the direct effect of the bacterial salt-tolerance functional pathway on the plant antioxidant system was 0.399 (P<0.01), while the chained indirect effect of dominant salt-tolerant bacteria–bacterial salt tolerance–plant antioxidant system pathway was 0.387, with a 95% confidence interval of 0.164-0.754 (**Supplementary Table 13**), suggesting a significant statistical mediating association between bacteria, bacterial salt-tolerant functions and plant antioxidant responses. Thus, salt stress was associated with the accumulation of osmotic adjustment substances in tiger nut plants and the concurrent enrichment of potentially halotolerant bacterial taxa in the rhizosphere. These enriched taxa were linked to bacterial salt-tolerance metabolic functions, which in turn were associated with plant antioxidant defenses and stress resistance traits.

In summary, moderate salt stress was associated with higher predicted abundances of metabolic pathways related to nitrification and nitrate reduction, which were linked to nitrogen- and sulfur-cycling functions. Potentially halotolerant bacterial taxa were correlated with soil nutrient cycling functions, and these functions were significantly positively correlated with plant stress resistance traits and the accumulation of plant underground dry matter. Among these pathways, the predicted abundance of KO_OS was most closely associated with plant osmotic adjustment substances and biomass. The Partial Least Squares Structural Equation Modeling (PLS-SEM) path analysis further identified plant osmotic adjustment substances and predicted bacterial metabolic functions as statistical mediators in the pathways linking salt stress to bacterial function, and potentially halotolerant bacteria to plant antioxidant systems, respectively.

## 4 Discussion

### 4.1 Salt stress enhances osmotic adjustment and antioxidant defense and shifts biomass allocation toward tuber development in tiger nut

Plants have evolved various defense mechanisms to cope with salt stress. This study found that, under salt stress, the contents of Pro, SS, and SP and the activities of SOD, POD, and CAT in the leaves of tiger nut significantly increased, all of which reached their highest levels under the CC3 treatment (soil salt concentration of 1.08 M; **Figures 1D–F**). The MDA content did not change significantly under low salt concentrations but significantly increased under the CC3 treatment, comparable to those under the CC4 and CC5 treatments (**Supplementary Figure 2C**). This is consistent with the physiological response characteristics of most plants in saline environments, the main reason for which is that excessive soluble salts in the soil reduce the water potential of the soil surrounding the root surface, thereby limiting the effective absorption of water by plants (van Zelm et al., 2020). To maintain cellular water uptake capacity, plant cells reduce their own cellular water potential by accumulating a large amount of osmoregulatory substances such as Pro and SS, thereby enhancing their competitive ability for soil moisture. Simultaneously, osmotic and ionic stresses caused by increased salt concentrations lead to the accumulation of toxic compounds such as reactive oxygen species (ROS), the disruption of cellular redox homeostasis, and membrane lipid peroxidation damage (Yang and Guo, 2018; Shi et al., 2025). To alleviate oxidative damage, the activities of antioxidant enzymes such as CAT and SOD are significantly enhanced in tiger nut cells in order to eliminate excess ROS. However, despite the defense system being maximally activated under the CC3 treatment, the degree of salt stress exceeded its carrying capacity; this resulted in the production of ROS exceeding the elimination rate, ultimately causing cell damage and a significant increase in MDA content.

In addition, this study found that the PH and RDW showed a downward trend in tiger nut with increasing salt concentration; in contrast, TDW increased with salt concentration, with the largest increase observed under the CC3 treatment. This phenomenon indicates that the tiger nut may adopt a “growth–defense” trade-off strategy under salt stress, potentially allocating limited photosynthetic products preferentially to the development of underground tubers. It is worth noting that, under the CC3 treatment, MDA accumulated significantly and TDW increased notably; this suggests that, when the tiger nut senses that the level of stress exceeds its own defense capacity, biomass allocation may be shifted toward tuber development. This shift in biomass allocation may represent a potential adaptive strategy of plants under adverse environmental conditions, and similar phenomena have been reported in halophytes such as *Suaeda salsa*, which allocated more photosynthetic products to reproductive organs under salt stress (Guo et al., 2019), and *Schrenkiella parvula*, an extreme halophyte model plant which flowers earlier under salt stress, thus also accelerating the transition from vegetative to reproductive growth (Tran et al., 2023).

In summary, tiger nut exhibits strong adaptability to salt stress. In particular, it adapts to salt-stressed environments by accumulating osmotic adjustment substances, enhancing the antioxidant defense system, and shifting biomass allocation toward tuber development.

### 4.2 Salt stress exerts differential effects on soil enzyme activities and suppresses microbial biomass accumulation

Soil extracellular enzymes are largely derived from microbial metabolism, playing a crucial mediating role in the carbon (C), nitrogen (N), phosphorus (P), and sulfur (S) cycles in terrestrial ecosystems (Liu et al., 2025b). They can serve as core indicators for the characterization of microbial activity and soil biogeochemical functions (Qu et al., 2025). This study found that, under salt stress, S_CAT activity significantly increased with increasing salt concentration, while URE and ALP activities showed an opposite trend; MBC, MBN, and MBP showed a decreasing trend with increasing salt concentration. This is consistent with the research of Hu et al. (2026), who concluded that increased soil salinity has a significant overall inhibitory effect on cellular enzyme activity. The activity of S_CAT continued to increase with increasing salt concentration, potentially representing the adaptive adjustment of soil microorganisms to eliminate reactive oxygen species (ROS) under salt stress. Salt stress reduces the diversity and ecological network complexity of bacterial communities carrying the *phoD* gene, weakening the ability of microorganisms to synthesize phosphatase. Furthermore, metal ions in saline environments are prone to forming insoluble phosphate precipitates, thus reducing substrate availability and collectively leading to a decrease in ALP activity (He et al., 2025). The activity of URE was significantly inhibited in this study, consistent with previous reports of salt stress reducing the soil nitrogen mineralization rate (Rietz and Haynes, 2003). The decrease in microbial biomass under salt stress is due to the fact that microorganisms require a large amount of energy to synthesize compatible solutes to resist osmotic stress, thus reducing the availability of resources needed for cell proliferation. Collectively, these results suggest the potential biochemical regulation strategies utilized by microorganisms to adapt to saline environments.

### 4.3 Salt stress was associated with a clear shift in the rhizosphere microbial community composition of tiger nut, with enrichment of functional groups possessing multiple stress-resistance traits

Salt stress can significantly affect soil microbial diversity and composition structure. In this study, it was found that the Shannon and Sobs indices showed a trend of first decreasing and then increasing with increasing salt concentration (**Figures 3A, B**), indicating that the alpha diversity of rhizosphere bacteria associated with tiger nut exhibited a nonlinear response to changes in salinity. This is consistent with the research results of Wang et al. (2025) on microeukaryotes in lakes with salinity gradients, which were mainly attributed to the environmental filtering effect of salinity. When salinity is increased from low-salt conditions, salt-sensitive bacteria are gradually eliminated and their diversity decreases; however, as salinity further increases, salt-tolerant bacteria are enriched through competitive release and niche differentiation, leading to recovered diversity (Rath et al., 2019; Menendez-Serra et al., 2022). The beta diversity analysis further indicated the dominant driving role of salinity on community structure (**Figure 3C**).

In terms of community composition, Firmicutes and Proteobacteria significantly increased under salt stress, and genera such as *Planococcus* and *Paracoccus* were significantly enriched (**Figure 3D**). This is similar to previous research findings, where bacterial communities in salt-stressed habitats were shown to tend to accumulate in the halophilic Proteobacteria phylum centered around *Halomonas* and the Firmicutes phylum dominated by *Bacillus* and *Planococcus* (Amechatte et al., 2026). Dai et al. (2025) also found that the salt-tolerant pioneer plants Suaeda glauca and Phragmites communis exhibit convergent enrichment of Firmicutes, Pseudomonas, *Bacillus*, *Planococcus*, and other salt-tolerant growth-promoting bacterial genera in their rhizospheres. Research has shown that *Halomonas* species can synthesize compatible solutes such as tetrahydropyrimidine, glycine betaine, and proline to maintain intracellular osmotic pressure. In particular, they use the Na^+^/H^+^ reverse transport system to eliminate excess sodium ions and secrete extracellular polysaccharides to alleviate ion toxicity (Miao et al., 2025). *Planococcus* species belong to the Firmicutes phylum and have a dense peptidoglycan cell wall that can reduce passive Na^+^ influx. They also have strong antioxidant enzyme activity, which helps to eliminate the reactive oxygen species produced under salt stress (Hassan et al., 2020). *Paracoccus* species can synthesize various compatible substances such as trehalose to resist osmotic stress, and also have heterotrophic nitrification–denitrification functions, allowing them to efficiently compete for nitrogen sources in environments disturbed by salt stress (Hollensteiner et al., 2023; Wu et al., 2024). *Allorhizobium*–*Neorhizobium*–*Pararhizobium*–*Rhizobium*, belonging to Rhizobiaceae, can synthesize osmoprotectants such as polyamines and have strong antioxidant enzyme activity. Some strains have nitrogen fixation potential, conferring metabolic advantages in nitrogen-limited stress environments (Diaz-Narvaez et al., 2024). Enrichment with these functional bacteria partially explains the phenomenon observed in this study of increased S_CAT activity under salt stress.

In summary, salt stress was associated with changes in bacterial community composition, alongside the enrichment of stress-tolerant bacterial taxa with stress-tolerance potential, including *Planococcus*, *Paracoccus*, and *Bacillus*. These microorganisms harbor the genetic potential for osmolyte synthesis, antioxidant defense, and ion homeostasis regulation. However, their actual functional contributions require further experimental validation.

### 4.4 Rhizosphere bacterial enrichment and plant salt-stress performance in relation to osmolyte accumulation

Plant osmoregulatory substances play crucial roles in microbial community remodeling. This study found that plant physiological factors have a much higher explanatory power for the structural variation in rhizosphere microbial communities in comparison to soil factors (**Figures 4A, B**). Among them, the independent contribution of plant osmotic regulators was greater than that of plant antioxidant enzyme systems, and was about 3.5 times that of soil factors (**Figure 4B**; **Supplementary Figure 5**). *Planococcus* and *Paracoccus* showed highly significant positive correlations with SS, SP, and Pro (**Figure 4C**). One possible interpretation of these correlations is that osmotic adjustment substances accumulated in tiger nut leaves under salt stress may also be released by roots and could potentially influence rhizosphere microbial community composition. This speculation is conceptually consistent with the plant “cry for help” strategy, which posits that plants selectively recruit beneficial functional microorganisms by secreting specific metabolites, such as sugars and organic acids, into the rhizosphere to enhance their adaptability to stress (Sasse et al., 2018; Zhalnina et al., 2018; Rolfe et al., 2019; Fang et al., 2025). Moreover, both *Planococcus* and *Paracoccus* reached their maximum relative abundance under the CC3 treatment, which coincided exactly with the peak accumulation of osmotic adjustment substances in tiger nut. This co-occurrence suggests a potential linkage between plant osmotic adjustment and the enrichment of these taxa. However, it is important to emphasize that we did not directly measure root exudates in this study, and leaf tissue concentrations cannot be directly equated with root exudation patterns. In addition, it should be noted that soluble protein was included in this study as part of an operational physiological index of osmotic adjustment, and its inclusion does not imply that total soluble protein represents a specific osmolyte. Therefore, the proposed role of osmotic adjustment substances as root-derived signals for microbial recruitment should be considered a hypothesis generated from correlation analysis, rather than an established mechanism. Future studies employing root exudate metabolomics, stable isotope tracing, and chemotaxis assays are needed to test this hypothesis directly.

Under salt stress, the predicted abundances of nitrogen-cycling-related functions showed significant differences. FAPROTAX prediction revealed that nitrification and denitrification functions were significantly higher under low- and medium-salinity treatments (CC1-CC3) (**Figure 5C**). This may be related to the significant enrichment of groups such as Paracoccus with nitrification and denitrification functions under this treatment (Hollensteiner et al., 2023). In addition, under this type of stress, microorganisms may accelerate their carbon metabolism turnover to maintain intracellular osmotic pressure and synthesize compatible solutes, which may, to some extent, be associated with the process of ammonia oxidation. The PICRUSt2 function prediction results indicated that KO-NIr was significantly enriched under the CC3 treatment (**Figure 5D**), which is consistent with the FAPROTAX results. In addition, under the CC3 treatment, the predicted abundances of KO_OS, KO_AS, and KO_IT were significantly higher. The Mantel test results showed that *Planoco*ccus was significantly positively correlated with CC and NC functions (q < 0.05), and that KO_OS, KO_AS, KO_IT, and KO_NIr were closely correlated with *Planococcus*, *Paracoccus*, CC, and NC functions (q < 0.05). These results suggest potential associations between predicted bacterial metabolic pathways and soil carbon cycling and nitrogen cycling functions, but the causative mechanisms require further experimental investigation.

This study found that *Planococcus* and *Paracoccus* were significantly positively correlated with TDW, SS, SP, Pro, SOD, and CAT in tiger nut (q < 0.05) (**Figure 4C**). Similarly, the predicted abundances of KO_OS, KO_AS, KO_IT, and KO_NIr were significantly positively correlated with TDW, SP, SS, Pro, and CAT in tiger nut (q < 0.05) (**Figure 5C**). The Mantel analysis results revealed that KO_OS, KO_AS, and KO_IT were significantly positively correlated with SS, Pro, SOD, and CAT in plants (**Figure 5E**). PLS-SEM path analysis further identified statistical mediation pathways linking plant osmotic adjustment to the salt-tolerant functional potential of rhizosphere bacteria, and linking potentially halotolerant bacteria to plant antioxidant systems (**Figure 5F**). These correlational patterns suggest an association between the predicted metabolic functions of rhizosphere bacteria and the stress-resistance traits of tiger nut, but the causative relationship requires further experimental verification. This speculation is consistent with previous reports in crops such as maize, soybean, and Populus euphratica, in which rhizosphere microorganisms exhibited a functional compensation mechanism under stress conditions, thus alleviating adverse effects on the host through significant upregulation of stress-resistance metabolic pathways (Ren et al., 2020; Li et al., 2024; Zheng et al., 2024; Pantigoso et al., 2025; Pang et al., 2026). However, direct evidence for such a compensatory effect in tiger nut induced by rhizosphere bacteria requires further validation through functional strain inoculation experiments. It should also be noted that the relatively small sample size (n = 18) and the complexity of the model resulted in suboptimal model fit indices (**Supplementary Table 8**), and some constructs showed discriminant validity limitations (**Supplementary Table 4**). Therefore, the mediation pathways identified by PLS-SEM should be interpreted as exploratory statistical associations rather than confirmed causal mechanisms. In future work, we will validate these findings with a larger sample size and further assess the predictive relevance of the model.

In summary, we proposed a conceptual framework for the potential synergistic salt tolerance in the association between tiger nut and root-associated bacteria. Specifically, salt stress was correlated with the accumulation of osmotic regulatory substances in tiger nut and the simultaneous enrichment of potentially halotolerant bacteria. Moreover, salt-tolerant functional pathways of bacteria were closely related to the stress-resistant metabolic traits of tiger nut plants. However, the mechanism underlying this potential synergy requires further confirmation through exogenous bacterial inoculation experiments. Future studies should also validate the PLS-SEM findings with a larger sample size. It should also be noted that soil salinity indicators such as EC, soluble cations and anions, exchangeable Na^+^, and SAR/ESP were not measured in this study. Given that salinity is the central experimental factor, future studies should incorporate these measurements to more comprehensively interpret the rhizosphere microbial response to salt stress.

## 5 Conclusion

In summary, tiger nut possesses a certain degree of salt stress resistance. Under salt stress conditions, plants shift biomass allocation toward tuber development, accompanied by significant accumulation of osmoregulatory substances and changes in the antioxidant defense spectrum, which may be associated with salt resistance. Among various treatments, 1.08 M NaCl caused the most significant physiological changes in tiger nut. Salinity is closely related to changes in the rhizosphere bacterial community of tiger nut. The alpha diversity of rhizosphere bacteria shows a U-shaped variation with the salinity gradient, and potentially halotolerant genera such as *Planococcus* and *Paracoccus* are significantly enriched. Plant osmoregulatory substances, such as soluble sugars (SS) and proline (Pro), are significantly correlated with the enrichment of potentially halotolerant bacterial communities. In addition, predicted bacterial metabolic pathways associated with salt stress exhibit a covariant relationship with soil nutrient cycling functions and plant stress resistance characteristics. PLS-SEM analysis showed that plant osmotic regulation factors exhibited a statistically mediated relationship between potentially halotolerant bacterial community structure and salt stress, and potentially bacterial salt tolerance function also exhibited a statistically mediated relationship between halotolerant rhizosphere bacteria and plant antioxidant systems. Overall, our dataset indicates covariation among plant physiological characteristics, rhizosphere bacterial composition, and bacterial salt tolerance metabolism under salt stress conditions. The salt tolerance of tiger nut may involve potential plant–microbe associations, which provide valuable references for improving crop yields in salt stress soils.

## Data Availability

The 16S rRNA sequencing data have been deposited in the NCBI Sequence Read Archive (SRA) under BioProject accession number PRJNA1498546 and are accessible at: https://dataview.ncbi.nlm.nih.gov/object/PRJNA1498546.
