# Supplementary material for: Salt stress shapes rhizosphere bacterial communities and is associated with salt tolerance in tiger nut (Cyperus esculentus L.)
Source: Front Plant Sci. 2026 Oct 29;17:1965745. doi: 10.3389/fpls.2026.1965745 (PMC13755316; doi:10.3389/fpls.2026.1965745)
Supplement: Supplementary file 1 [file DataSheet1.docx]

Supplementary Material

# 1 Supplementary Figures and Tables

## 1.1 Supplementary Figures

**Supplementary Figure 1.** Rarefaction curves of the Sobs index for all samples. The x-axis represents the number of reads sampled, and the y-axis represents the observed number of OTUs CK, control group (0 M NaCl); CC1-CC5, salt treatment groups with increasing NaCl concentrations.

**Supplementary Figure 2.** Analysis of root fresh weight, tuber fresh weight, leaf malondialdehyde content, and SPAD of tiger nuts under different salt concentrations. (A) root fresh weight (RFW), (B) tuber fresh weight(TFW), (C) malondialdehyde (MDA), Relative chorophyll content (SPAD).CK, control group (0 M NaCl); CC1-CC5, salt treatment groups with increasing NaCl concentrations. Bar charts represent mean ± SD. Different lowercase letters above the bars indicate significant differences among treatments (one-way ANOVA followed by Tukey–Kramer test, P<0.05).

**Supplementary Figure 3.** Soil sucrase activity under different salt concentrations.CK, control group (0 M NaCl); CC1-CC5, salt treatment groups with increasing NaCl concentrations. Bar charts represent mean ± SD. Different lowercase letters above the bars indicate significant differences among treatments (one-way ANOVA followed by Tukey-Kramer test, P<0.05).

**Supplementary Figure 4.** Analysis of dominant bacteria in the rhizosphere of tiger nuts under different salt concentrations. (A)The top 10 bacterial phyla, (B)Top 20 bacterial genera.

**Supplementary Figure 5.** Variance decomposition analysis of the effects of plant stress resistance and soil traits on the rhizosphere bacterial community structure of tiger nut. P plant stress resistance, S soil traits.

**Supplementary Table 1** Summary of 16S rRNA gene sequencing data for all samples.

**Supplementary Table 2** Grouping basis and functional annotation of KOs assigned to the KO_OS, KO_AS, KO_NIr, and KO_IT categories.

**Supplementary Table 3** Measurement model assessment: Outer loadings, Cronbach’s alpha, composite reliability, and AVE.

**Supplementary Table 4** Discriminant validity: Heterotrait-monotrait ratio (HTMT) list.

**Supplementary Table 5** Collinearity diagnostics: Variance inflation factor (VIF) for inner model. paths

**Supplementary Table 6** R-square overview of endogenous latent constructs in the PLS-SEM model.

**Supplementary Table 7** f-square effect sizes of paths in the PLS-SEM model.

**Supplementary Table 8** Model fit indices.

**Supplementary Table 9** Correlation analysis between dominant bacterial genera in the rhizosphere of tiger nuts and plant and soil indicators, with Benjamini-Hochberg FDR-adjusted P values.

**Supplementary Table 10** Mantel test analysis linking salt-resistance KO pathways, keystone bacterial genera, and soil ecosystem functions under saline stress, with Benjamini-Hochberg FDR-adjusted P values.

**Supplementary Table11** Correlation analysis between salt-tolerant KO pathways, soil ecological functions, soil indicators, and plant indicators, with Benjamini-Hochberg FDR-adjusted P values.

**Supplementary Table 12** Path coefficients of the partial least squares structural equation model (PLS-SEM).

**Supplementary Table 13** Specific indirect path coefficients and 95% bootstrap confidence intervals for the salt tolerance regulatory model of tiger nut.
